# Supplementary material for: Feasibility and acceptability of research-grade wearables for health and labor capacity monitoring in the context of climate change and heat stress: The case of Nouna, Burkina Faso
Source: PLoS One. 2025 Oct 15;20(10):e0330835. doi: 10.1371/journal.pone.0330835 (PMC12527170; doi:10.1371/journal.pone.0330835)
Supplement: S1 — (DOCX) [file pone.0330835.s001.docx]

**Entry Survey**

**Date:**

**Time:**

**Place:**

**Interviewer:**

**Respondent’s ID:**

**NOTE for the Interviewer:** A description of participant’s home characteristics and family members must be included in the overview questionnaire, if not retrievable from HDSS

1. **Demographics (to be anonymized before sharing data with unauthorized individuals, just needed to be linked with HDSS repository)**

**A1**. What is your first name?

**A2.** What is your middle name?

**A3.** What is your last name?

**A4.** What is your mother's first name?

**A5.** What is your mother's name?

**A6.** What is your mother's last name?

**A7.** What is your father's first name?

**A8.** What is your father's name?

**A9.** What is your father's last name?

**A10.** What is the name of the Village or town where you are currently a resident? _____

**A11.** In which county is the village in A10 above situated? ______________

**A12.** When did you start living in the village mentioned in A10 above for your current residency episode? (YY/MM/DD):

**A13.** Date of birth (YY/MM/DD): _______

**A14.** Age (in years): ______

**A15.** Age group: 🗖_1_ 20-30 / 🗖_2_ 30-40 / 🗖_3_ 40-50 / 🗖_4_ 50 and above

**A16.** Sex: 🗖_1_ Male / 🗖_2_ Female/ 🗖_3_ Intersex / 🗖_4_ Indeterminate / 🗖_5_ Other

**A17.** Education (highest level attained): 🗖_1_ None / 🗖_2_ Primary / 🗖_3_ Secondary / 🗖_4_ Higher Secondary / 🗖_5_ University / 🗖_6_ Refuse to answer

**A18.** Smoking: 🗖_1_ Smoker /🗖_2_ Non-Smoker /🗖_3_ Ex-smoker/ 🗖_4_ Refuse to answer

**A19.** Consuming alcohol: 🗖_1_ Yes 🗖_2_ No 🗖_3_ Ex/ 🗖_4_ Refuse to answer

**A20.** Any existing illness: 🗖_1_Diabetes 🗖_2_Hypertension 🗖_3_Respiratory illness, specify 🗖_4_Others, specify 🗖_5_ Refuse to answer

1. **General overview of your working experiences**

**B1.** In the past two years, what occupational activity was your **main source of income**?

(Choose one)

1. working in the field (agriculture, fishing, grounds maintenance, etc.)
2. selling goods at the market, roadside, streets, or shop
3. household chores (cooking, cleaning, child-care, etc.)
4. skilled labor (carpentry, tailoring, plumbing, electricals, driver etc.)
5. professional job (teaching, nursing, secretarial etc.)
6. Student (learning)
7. Other, specify______________
8. Refuse to answer

**B2.** In which environment did you carry out this activity (relating to temp)?

1. Outdoor / b. Indoor / c. Both

**B3.** Have you noticed any tendency towards higher heat exposure during recent years compared to 20-30 years ago (assuming you were around then)? ____________

**B4.** How long did you work in the activity mentioned in B1 above ______years/months (more than 6 months means acclimatized)?

**B5.** How many hours per day do you usually work excluding regular break timings? _____

**B6.** Have you ever been affected by a heat related illness during you working activity?

**B7.** Have you ever taken sick leave/permission due to heat?

🗖_1_ Yes / 🗖_2_ No/ 🗖_3_ I don’t know / 🗖_4_ Refuse to answer

If yes, approx. how many hours/days in a week/month? _________________

**B8.** Have you lost any wages due to absenteeism in the dry season? 🗖_1_ Yes / 🗖_2_ No

If yes, how much __________________________?

**B9.** Have you ever been advised/ ever been admitted in hospital/medical center to take off due to heat related illness? 🗖_1_ Yes / 🗖_2_ No If yes, approximately how many days_____

**B10.** How does heat affect other aspects of your work (during hot seasons)?

🗖_1_ Absenteeism/ 🗖_2_ Less productivity/ 🗖_3_ Irritation/Interpersonal issues/Work related issues with manager/ 🗖_4_ Take more time to complete same task

**B11.** Have you been advised to take time off from work due to heat related illness?

🗖1 Yes / 🗖2 No/🗖_3_ I don’t know / 🗖_4_ Refuse to answer

**B12.** Have you been admitted in hospital/medical center due to heat related sickness?

🗖1 Yes / 🗖2 No/🗖_3_ I don’t know / 🗖_4_ Refuse to answer. If yes, approximately how many days_____

**B13.** Have you been admitted in hospital/medical center due to dehydration?

🗖1 Yes / 🗖2 No/🗖_3_ I don’t know / 🗖_4_ Refuse to answer. If yes, approximately how many days_____

**B14.** Have you ever been admitted to hospital/medical center because of kidney problems? 🗖_1_ Yes / 🗖_2_ No/🗖_3_ I don’t know / 🗖_4_ Refuse to answer. If yes: Approximately for how many days? ______________

**B15.** Have you ever injured yourself at work during very hot weather?

🗖_1_ Yes / 🗖_2_ No/🗖_3_ I don’t know / 🗖_4_ Refuse to answer. If yes: What kind of health. problems?___________________

1. **General overview in relation to heat exposure at home**

**C1.** Are you comfortable with the ambient temperature at home? 🗖_1_ Yes / 🗖_2_ No

**C2.** Is heat exposure a problem during the hot season? 🗖_1_ Yes / 🗖_2_ No

**C3.** How many months do you feel hot /uncomfortably hot at home?

🗖_1_ 1-3 months/ 🗖_2_ 4-6 months/ 🗖_3_ 7-9 months / 🗖_4_ 9-12 months/ 🗖_5_ Never

**C4.** Specify which months are the worst _______________________

**C5.** Describe how bad the heat stress can be in the hot season

🗖_1_ Extremely bad/ 🗖_2_ Very bad/ 🗖_3_ Bad/ 🗖_4_ Manageable/ 🗖_5_ No stress at all

**C6.** Is the building, where you spend most of your time at home, built and equipped to reduce heat stress? (building style, location in relation to shade trees, air conditioning, etc.)

**C7.** In your opinion, to what extent were your daily chores, e.g. cleaning and washing clothes, affected during the hottest days, in the past year?

1. Very little 2. Little. 3. Much 4. Very much 5. Not sure

**C8.** Was your health affected by heat at home, in the past year?

🗖_1_ Yes / 🗖_2_ No/🗖_3_ I don’t know

**C9.** Indicate whether you **used to** live in the past two years with any of the following individuals who may be vulnerable to increased temperature at home?

1. children, 2. women, 3. elderly 4. Other, specify _______________ 5. I live alone

**C10.** Describe the means by which the people **usually** (in your experience) manage to reduce any heat effects (air conditioning, doing activities at night, etc.)?_________

1. **Questions for women only**

**D1.** How old were you when you had your first period (menstruation)? If you aren’t sure, please try to estimate this. _______________

**D2.** How old were you the first time your period stopped for a whole year? (Do not include times when your period stopped because of pregnancy, breastfeeding, or using birth control (for example, an IUD)? ________ years old or. □ Doesn’t apply (periods haven’t yet stopped for a whole year)

**D3**. Have you given birth to one or more children?

No ➔ Go to Question D5

Yes

**D4.** How many children have you had? _________ children

**D5.** Do you currently use contraceptive medication? (birth control pill)? 🗖_1_ Yes / 🗖_2_ No

**D6.** How heavy is your menstrual flow usually?

1. Light
2. Moderate
3. Heavy (clots/flooding)
4. Can’t remember
5. Refuse to answer

**D7.** Do you experince any of the following symptoms when you have a period?

Please tick all that apply

1. Pelvic pain (pain in the lower party of your belly)
2. Pain on opening your bowels
3. Bleeding from your back passage when opening your bowels
4. Pain on passing urine
5. Passing blood in your urine
6. Lower back pain
7. Pain in upper leg or thighs
8. Nausea
9. Tiredness
10. Refuse to answer

**D8.** Does have menstrual problems ever interfered with your work, travel or social life?

_1_Yes / _2_No/ 🗖_3_I don´t know / 🗖_4_ Refuse to answer
