# Supplementary figures and images for: Feasibility and acceptability of research-grade wearables for health and labor capacity monitoring in the context of climate change and heat stress: The case of Nouna, Burkina Faso
Source: PLoS One. 2025 Oct 15;20(10):e0330835. doi: 10.1371/journal.pone.0330835 (PMC12527170; doi:10.1371/journal.pone.0330835)

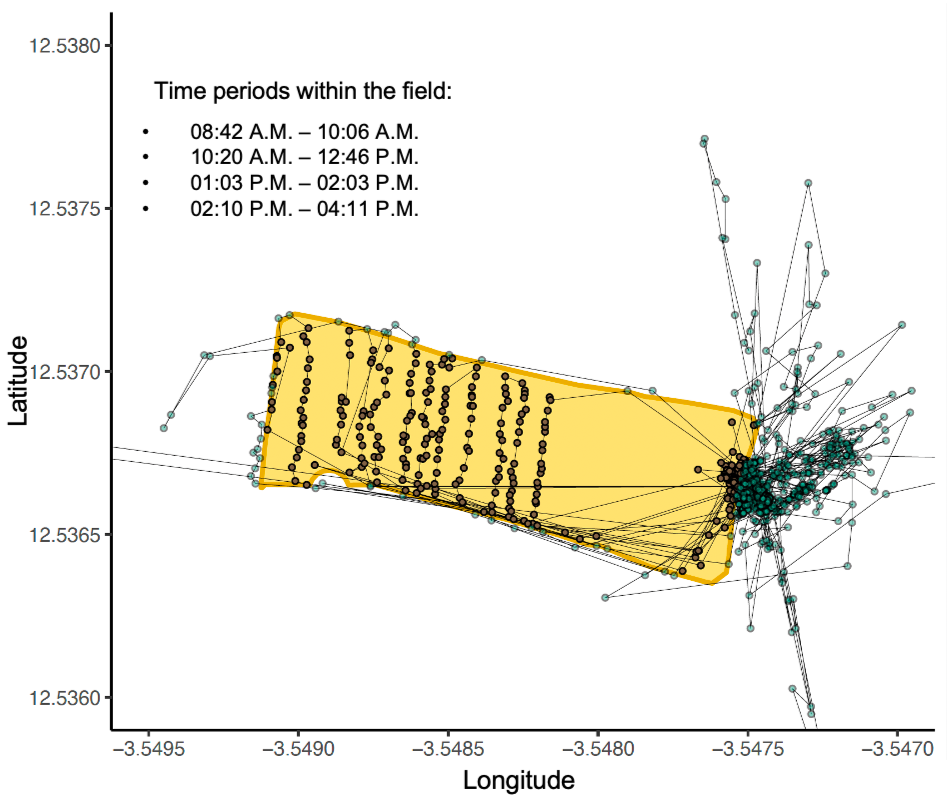

Supplement: S2 Fig — The total time spent in the field by the subject was 433 min, the total distance covered within the fields’ boundaries was 3339 m. The recording was done on the 23rd of October 2021, between 0 A.M. (00:00:24) and 0 P.M. (23:59:19). During this time span the subject was 4 times within the fields’ boundaries. (TIFF) [file pone.0330835.s002.tiff]
