# Supplementary material for: Feasibility and acceptability of research-grade wearables for health and labor capacity monitoring in the context of climate change and heat stress: The case of Nouna, Burkina Faso
Source: PLoS One. 2025 Oct 15;20(10):e0330835. doi: 10.1371/journal.pone.0330835 (PMC12527170; doi:10.1371/journal.pone.0330835)
Supplement: S3 — (DOCX) [file pone.0330835.s003.docx]

**ACCEPTABILITY QUESTIONNAIRE**

**Participant’s ID**

__________________

**Appointment’s Date**

__________________

**Blood pressure**

__________________

**Anthropometry and body composition**

Please report here the values of BIA:

Resistance (R): ________

Reactance (xc): ________

Weight (kg) _________

Height (kg) ________

As the questionnaire was conducted digitally, the identical questions were inquired separately for each device, being: **Faros** (ECG) **Tcore** (dual-heat-flux thermometer and data logger)

**GENEActiv** (Accelerometric wrist-worn band) **GPS** (data logger)

Several answering statements were provided for each question, and participants had to select a number on the Likert Scale reflecting their agreement towards the statement.

**Likert Scale: Strongly disagree = 1, Disagree = 2, Undecided = 3, Agree = 4, and Strongly agree = 5.**

Additionally, each question allowed respondents to describe specific impressions/experiences in free text or add further comments.

Single-select, multiple-select, and open-ended questions are specified as such.

## Overall impressions and experiences:

1. **What were your first impressions of the sensor?**
   1. I was happy to wear the sensor
   2. I was disturbed by wearing the sensor
2. **I found the sensor:**
   1. Easy to use
   2. I like it
   3. Interesting
   4. Important for my health
   5. Requiring too much attention and care
   6. Useful
   7. Annoying
   8. Disturbing
   9. Difficult
   10. Cumbersome
   11. Strange
   12. Without sense
   13. Other (describe)
   14. Further comments
3. **How do you feel about having different monitoring you at once?**
   1. Easy to use
   2. I like it
   3. Interesting
   4. Important for my health
   5. Requiring too much attention and care
   6. Useful
   7. Annoying
   8. Disturbing
   9. Difficult
   10. Cumbersome
   11. Strange
   12. Without sense
   13. Other (describe)
   14. Further comments
4. **How did you find using the sensor?**
   1. Easy to use
   2. I like it
   3. Interesting
   4. Important for my health
   5. Requiring too much attention and care
   6. Useful
   7. Annoying
   8. Disturbing
   9. Difficult
   10. Cumbersome
   11. Strange
   12. Without sense
   13. Other (describe)
   14. Further comments
   15. Here participants are presented with specific experiences they might have had with specific sensors likely to be more cumbersome (Tcore and ECG):
       1. **Tcore:**
          1. Was the Tcore headband uncomfortable?
          2. Was there a requirement to frequently adjust the headband?
          3. Did you feel increased heat on the head because of the headband?
          4. Did a displacement of the Tcore sensor happen frequently?
          5. Others (describe)
       2. **Faros:**
          1. Was the Faros ECG uncomfortable?
          2. Did you need to fix or reposition the electrodes frequently?
          3. Did the Faros ECG frequently fall off?
          4. Did you use new electrodes because they fell of or were not sticking well?
          5. Did the cables disturb you or limit your work?
          6. Others (describe)
5. **What did you like/dislike?**
   1. Good Usability (easy to use)
   2. Right (i.e., appropriate, convenient) Weight
   3. Right (i.e., appropriate, convenient) Size
   4. Nice Appearance
   5. Convenient Positioning
   6. Comfortable to wear
   7. Bulky
   8. Too big
   9. Difficult to wear
   10. Disturbing
   11. Other (describe)
   12. Further comments

## Specific issues:

1. **Did you experience any issues/problems by wearing this sensor?**This is a single-select question:
   1. Yes
   2. No
   3. I do not know

If answered with YES, participants were asked multiple select from the following list:

- 1. Cutaneous rush
  2. Itch
  3. Sensor causing pain
  4. Limitation of movements
  5. Working disturbances
  6. Sleep disturbances
  7. Disturbances of your personal care daily routine (personal hygiene)
  8. Must frequently check and take care of the sensor
  9. Others (describe)
  10. Further comments

## Influence on daily activities, work and sleep:

1. **How did the sensor influence your working/daily activity?**
   1. It influenced my working/daily activity
   2. I forgot I was wearing it
   3. It was not disturbed
   4. Others (describe)
   5. Sometimes difficult
   6. Requiring time /attention/care
   7. I interrupted my activities several times because of the sensor
   8. I had to remove the sensor
   9. The sensor was limiting my movements
   10. I had pain or adverse reactions (like cutaneous rush, itch)
   11. The sensor was stuck on my skin because of sweating
   12. I felt increased heat because of wearing the sensor
   13. Others (describe)
   14. Further comments
2. **Did wearing the device have any effects on your sleep?**
   This is a single-select question:
   1. Yes
   2. No
   3. I do not know

If answered with YES, participants were asked multiple select from the following list:

- 1. Sometimes I woke up
  2. I woke up frequently
  3. I could not sleep at all because of the sensor
  4. I felt tired in the morning (poor sleep quality)
  5. The sensor fell off during the night
  6. I felt increased heat because of wearing the sensor
  7. Others (describe)
  8. Further comments

## Social acceptance:

1. **Did you have any reason to remove the sensor?**This is a single-select question:
   1. Yes
   2. No
   3. I do not know

If answered with YES, participants were asked multiple select from the following list:

- 1. Limiting my activities
  2. Adverse reactions
  3. Others (describe)

1. **Were you comfortable wearing the sensor in public?**This is a single-select question:
   1. Yes
   2. No
   3. No answer
2. **Did people ask you about the sensor?**

This is a single-select question:

- 1. Yes
  2. No
  3. I do not know

1. **Did the sensor become a topic of conversation?**

This is a single-select question:

- 1. Yes
  2. No
  3. I do not know

1. **Please describe what other people say to you about the sensor.**
   This is an open-ended question; participants were asked to answer in free speech.

## Summary:

1. **In summary, did wearing devices cause a change in your usual habits, and if so, how?**
   This is a single-select question:
   1. Yes
   2. No
   3. I do not know

If answered with YES, participants were asked multiple select from the following list:

- 1. Limiting my social activities
  2. Limiting my working activities
  3. Affecting my sleep
  4. Scarce social acceptance
  5. Adverse physical reactions
  6. Others (describe)

If answered with YES, participants were asked to describe what would need to change to develop the habit of using devices or engaging with them (Open-ended question)

## Hindering factors:

1. **If you had to wear this sensor for a longer time period, for example, months, what would be the barriers to participating in this trial for you?**This is a multiple-select question:
   1. Length of necessary time to wear
   2. Interaction level required (i.e., taking care of the sensor)
   3. Desired sensor feedback (i.e., get information from the sensor)
   4. Adverse effects
   5. Social acceptance
   6. Disturbance daily activity
   7. Disturbance sleep
   8. Disturbance of personal hygiene routine
   9. Others (describe)
   10. Further comments
