# Supplementary material for: Feasibility and acceptability of research-grade wearables for health and labor capacity monitoring in the context of climate change and heat stress: The case of Nouna, Burkina Faso
Source: PLoS One. 2025 Oct 15;20(10):e0330835. doi: 10.1371/journal.pone.0330835 (PMC12527170; doi:10.1371/journal.pone.0330835)
Supplement: Attachment — Submitted filename: Response to Reviewer 2.docx [file pone.0330835.s009.docx]

**Reviewer comment:**
This is a valuable report that discusses the potential of wearable devices in the harsh environment of Africa.
However, there are a number of places throughout the report where the description is insufficient.
In addition, there are some places where the experimental methods and interpretation of the results are not fully explained.
Please consider making revisions, focusing on the points we have commented on.

**Authors response:**

We thank you for your insightful and constructive commentary, which has been invaluable in refining our work. We acknowledge that some areas of our report required more detailed descriptions and clearer explanations of our experimental methods and results interpretation. In response, we have thoroughly revised these sections to provide additional context and clarity, ensuring that our methodology and findings are more comprehensively presented and reproducible. We addressed the raised issues and adjusted the manuscript processing accordingly as presented in detail in the table above. Our revisions include expanded details on the data collection process, adjustments in data processing and analysis, and a more rigorous discussion of our methodology and the implications of our results. We are confident that these improvements enhance the overall clarity and scientific rigor of our report. Thank you again for your thoughtful feedback, which has significantly contributed to the revision process and general quality of our manuscript.

| **Reviewer comment** | **Authors response** |
| --- | --- |
| L.25: You write ‘mean ambient temperatures of 1.5°’, but the unit of temperature should be ‘°C’, not ‘°’. | Thank you for pointing this out, we have adapted this accordingly. |
| L.64: You mention‘even moderate heat stress (18° WBGT)’, but WBGT 18°is really ‘moderate heat stress’? In the reference cite, it seems to be mild heat stress. Also, 18° and 40° are inappropriate units, and should be °C. | Thank you very much for bringing this to our attention. We agree that the terminology must be adjusted accordingly and have done so in the manuscript. Please see L. 65. To clarify, we chose the reference in question as the authors present novel insights on heat stress, especially so-called mild heat stress, which was formerly considered to have at best minimal effects on physical work capacity. The authors juxtapose their findings to established thresholds previously published, for example by Kjellström et al., and argue that heat stress even at as low as 18°C WGBT has been previously underestimated regarding its effect on the reduction of physical work capacity. |
| L89: Why is PSI not used in this study, even though it is mentioned as an excellent indicator here? If possible, please mention it | Thank you very much for this suggestion. We do agree that this is an important consideration. Our overarching goal is to create a platform for future investigations into occupational heat stress, including the adaption of indices such as PSI to ultimately enable the development of preventive and adaptive strategies. To demonstrate the feasibility and suitability of our methodological approach for such a goal, we added the calculation of PSI to our manuscript. We based the calculations on the works of Davey et al. and Byrne et al. Please see L. 253-257. |
| L.153-156, L.172-173: Please clearly state the names of the devices used in the main text, not just in Figure | Thank you for addressing this. The names of the implemented devices are cited later in the methods section of the manuscript within the sections dedicated to monitoring. We indeed checked once again on the completeness of the stated information and if needed adjusted it accordingly. Please see L. 200, 207-207, 213, 218. |
| lso, please describe how the portable WBGT meter used here calculates WBGT.  The method specified in ISO 7243 is the only correct method for WBGT, and if a different method is used, it is considered to be nothing more than an estimate, so you need to show in the literature, etc., how the estimate was made. Otherwise, you should indicate that these values are merely estimates. | Thank you very much for bringing this to our attention. As suggested, we have closely checked the portable hygrometer implemented in this feasibility study and the formula it employs to estimate WBGT. It does align with the method specified in ISO 7243 for WBGT estimation without solar load (WBGT = 0.7 × Tₙwᵦ + 0.3 × Tg). To improve the clarity of our manuscript we adjusted the according part of the manuscript by mentioning the ISO formula for WBGT estimation and stating the alignment of our implemented hygrometer. Please see L. 193-196. |
| L170-172: What measurement factors were used to estimate WBGT in this measurement system, and how was it estimated? Please specify this in the main text so that it is clear to those who have only read this paper, rather than just citing reference 28. | We appreciate your feedback, which has been instrumental in refining our work. The automated weather station we implemented in our research measured several variables, including air temperature, relative humidity, precipitation, wind speed and direction, and global radiation. As the weather station does not include a black globe, WBGT was estimated according to Carter et al. by incorperating the natural wet bulb temperature (T_nwb_), solar radiation (SR), relative humidity (RH), and air temperature (T_a_):  WBGT= 0.7 × T_nwb_ + 0.2 × [0.009624*SR + 1.102 × T_a_ – 0.00404 × RH – 2.2776] + 0.1 × T_a_  To clarify the flow of reasoning within our manuscript, we specified this and included the above-mentioned formula into the text. Please see L. 181-189. |
| In addition, I have looked at Reference 28, and while there is a certain degree of correlation between the methods for estimating WBGT from meteorological observation data, they are not yet established, and it is thought that more data is needed. It is thought that WBGT is correct when measured using the method specified in ISO 7243, and that the accuracy of WBGT measured using other methods needs to be considered. | Thank you for your constructive critique. We adopted the approach described by Carter et al. to estimate WBGT using automated weather station data without from a weather station without a black globe. We recognize that the method specified in ISO 7243, which involves direct measurement with specialized WBGT monitors for outdoor WBGT estimation, is considered the gold standard. However, the method we used provides a cost‐effective and practical alternative for many applications, particularly for risk mapping and large‐scale studies. Carter et al. demonstrated that WBGT estimates derived from proxy data are strongly correlated with measurements from commercial WBGT monitors. Therefore, this approach provides the best proxy to estimate WBGT for outdoor data in our given setting. We agree further validation across diverse environments and more extensive datasets would be beneficial. However, the method has proven sufficiently accurate for the purpose of our study. Nonetheless, we have now clearly noted its limitations relative to ISO 7243 and other estimation methods for outdoor WBGT in our revised discussion. Please see L. 527-546. |
| L.175: Fig. S3 contains technical information on environmental measurements, but this information is completely inadequate. As mentioned above, please clearly state what measurements were taken and how the WBGT value was calculated (or estimated). | Thank you for your comment. We now provided specifications of the portable hygrometer. It is in line with ISO 7342 for WGBT estimation without solar load. We now further provide a detailed description of the automated weather station in use and the approach which was used to estimate WGBT based on the work of Carter et al. To be more concise and not confuse the readers we decided to provide this information primarily in the manuscript text and therefore removed Fig S3. Please see L. 193-196. |
| L177-178: You use a wrist-mounted accelerometer, but can you understand the activity status of the whole body with a wrist-mounted accelerometer? I think it would be better to cite any references you have on this point. | Thank you for bringing this up, we acknowledge your concerns. However, wrist-worn accelerometry is considered a feasible and validated approach in the field of physical training and physical fitness level assessments across a range of activities, as shown for example by Eslinger et al. for the GENEActiv accelerometer. To find the reference in our manuscript, please refer to L.202.   - Esliger DW, Rowlands AV, Hurst TL, Catt M, Murray P, Eston RG. Validation of the GENEA Accelerometer. Med Sci Sports Exerc. 2011 Jun;43(6):1085-93. doi: 10.1249/MSS.0b013e31820513be. PMID: 21088628. |
| L180: Please cite the R package used and also the references for each calculated value. | Thank you for your comment. In our revision process we opted to streamline our manuscript and to make it more concise and reflective of its original intent, namely exploring technical feasibility and social acceptability. Therefore, we decided to not include derived calculations such as AEE and step count in this revised version. |
| L183: There is a missing period after ‘during nightly rest [30,31]’. | Thank you for your comment, the text has been adjusted accordingly. |
| L185-188: The deep body temperature is measured using a heat flux sensor attached to the forehead, but there is a lack of information about the validity of this. You cite reference 33, but this reference only measures CBT in the same way as this report, and does not demonstrate its validity. It seems that the paper cited in reference 33 should be cited, not reference 33? Please re-examine the validity of the cited references. | Thank you for your helpful insights. We acknowledge the novelty of the dual heat-flux approach to estimate core body temperature. However, there is a growing body of work showing promising results regarding its applicability. The Tcore double sensor has been originally developed jointly with Dräger and Koralewski as prototype in the frame of the ESA/DLR sponsored study “Circadian Rhythm”. The reasoning was to enable continuous and non-invasive core body temperature monitoring in astronauts on the International Space Station. There, it has been successfully implemented for more than 10 years. The new and upgraded version of the Tcore double sensor, which is the one we implemented in our study, is furthermore currently in use in German hospitals, especially in Intensive Care Units. We added the according references to our manuscript, please see L. 212.   - Gunga HC, Werner A, Stahn A, Steinach M, Schlabs T, Koralewski E, Kunz D, Belavý DL, Felsenberg D, Sattler F, Koch J. The Double Sensor-A non-invasive device to continuously monitor core temperature in humans on earth and in space. Respir Physiol Neurobiol. 2009 Oct;169 Suppl 1:S63-8. - Mendt S, Maggioni MA, Nordine M, Steinach M, Opatz O, Belavý D, Felsenberg D, Koch J, Shang P, Gunga HC, Stahn A. Circadian rhythms in bed rest: Monitoring   core body temperature via heat-flux approach is superior to skin surface temperature Chronobiol Int. 2017;34(5):666-676. doi: 10.1080/07420528.2016.1224241.   - Janke D, Kagelmann N, Storm C, Maggioni MA, Kienast C, Gunga HC, Opatz O. Measuring Core Body Temperature Using a Non-invasive, Disposable Double-Sensor During Targeted Temperature Management in Post-cardiac Arrest Patients. FrontMed (Lausanne). 2021 May 5;8:666908. doi:10.3389/fmed.2021.666908. - Soehle M, Dehne H, Hoeft A, Zenker S. Accuracy of the non-invasive Tcore™ temperature monitoring system to measure body core temperature in abdominal surgery. J Clin Monit Comput. 2020 Dec;34(6):1361-1367. doi: 10.1007/s10877-019-00430-9. Epub 2019 Nov 26. PMID: 31773375. |
| Furthermore, it is our understanding that the estimation of deep body temperature using this method is not yet established, and there is debate about its validity. I think this point should be mentioned in the discussion. | Thank you for your constructive critique. Though we acknowledge the relative novelty of the dual heat-flux method for core body temperature monitoring, to our best understanding it has been validated multiple times against rectal thermistors or rectal temperature probes. Validation studies have been conducted in both clinical, and outdoor settings. It is true, that monitoring via the dual heat-flux method may not provide the exact core body temperature as compared to direct esophageal measurements. Nonetheless, it is still a very convenient and practical method for 24-hour continuous core body temperature monitoring in a naturalistic environment. Especially in our setting the ability to observe physiological responses to environmental stressors and internal heat load changes without stark interference in our participant’s daily routine, is of paramour importance. The dual heat-flux method provides that in a reliable manner. We have added two references regarding the validation of the Tcore sensor. To clarify, we have adapted the discussion of our manuscript, please refer to L. 475-487.   - Zeiner S, Zadrazil M, Willschke H, Wiegele M, Marhofer P, Hammerle FP, Laxar D, Gleiss A, Kimberger O. Accuracy of a Dual-Sensor Heat-Flux (DHF) Non-Invasive Core Temperature Sensor in Pediatric Patients Undergoing Surgery. J Clin Med. 2023 Nov 9;12(22):7018. doi: 10.3390/jcm12227018. PMID: 38002632; PMCID: PMC10672443. - Tsadok I, Scheinowitz M, Shpitzer SA, Ketko I, Epstein Y, Yanovich R. Assessing rectal temperature with a novel non-invasive sensor. J Therm Biol. 2021 Jan;95:102788. doi: 10.1016/j.jtherbio.2020.102788. Epub 2020 Nov 17. PMID: 33454029. |
| L188-189: Please either provide details of the ‘custom-made headband’ or a list of references. | We appreciate you mentioning this. The headbands we used in our study were designed as prototypes by our research unit specifically for the implemented Tcore CBT sensor and HFMU data logger to improve wearability and comfort for our participants. As the headbands were designed explicitly for research purposes within our research unit frame, there are no prior references, apart from our previously published study protocol paper, which we included in our reference list.   - Barteit S, Boudo V, Ouedraogo A, Zabré P, Ouremi L, Sié A, Munga S, Obor D, Kwaro D, Huhn S, Bunker A, Sauerborn R, Gunga HC, Maggioni MA, Bärnighausen T. Feasibility, acceptability and validation of wearable devices for climate change and health research in the low-resource contexts of Burkina Faso and Kenya: Study protocol. PLoS One. 2021 Sep 30;16(9):e0257170. doi: 10.1371/journal.pone.0257170. PMID: 34591893; PMCID: PMC8483291. |
| L193: Please include information that will allow readers to reproduce ‘the native software’. | Thank you for the remark. It is of great importance to us, to ensure the reproducibility of our approach. Therefore, we adjusted our data processing and the according information in this revised manuscript. We now provide two R-packages we implemented instead, that can be freely downloaded and implemented with little to no barriers. We used the Pan-Tompkins algorithm and implemented in the *rpeaks* package for R-peak detection and the *RHRV* package for heart rate extraction. In our udated text we included all the necessary references for the R-packages in use Please refer to L. 248-249. |
| L197-198: We have checked the WBGT device, and it appears to be a product made by Canmore in Taiwan, not Germany. Please be more precise in your description.  https://asset.conrad.com/media10/add/160267/c1/-/en/001217431ML03/manual-1217431-renkforce-gp-102-g-porter-gps-logger-blue-black.pdf | Thank you for your remark. We appreciate your insightful comment, as it helps heighten the precision of our manuscript. After checking the provided information, we adjusted the information accordingly. Please refer to L. 218. |
| L214-218, L269-271: The quality of data is evaluated by asking whether ‘all possible data points exist in the valid data set’, but is this enough to determine validity?  If the accuracy of the data is not evaluated, it is difficult to evaluate the quality, isn't it?  Please include your thoughts on this point in your discussion. | We sincerely appreciate your careful review, as it helped to streamline and focus our research work. During the revision process, we shifted our focus to appropriately demonstrate the primary aim of our study, namely the feasibility of equipping a rural population in SSA with wearables for a prolonged time period. We adjusted our data processing and analysis, accordingly, now evaluating data completeness and, if possible, data quality. We decided to primarily focus on data completeness and quality for HR and CBT data, as there are established cut-off values, we were able to implement for quality assessment. For ACT data, which is a continuous accelerometric data stream and not directly physiological data with established cut-off values for excluding outliers, we focused on data completeness. Here we decided to declare data points valid/complete, if the GENEActiv has truly been worn during measurement, which we determined by analyzing the near-body temperature it records. We applied the same reasoning to GPS data, focusing primarily on data completeness, as it is also not physiological data with established cut-off values. For our adjusted methodology please see L. 259-267. |
| L246: The data integrity is said to have reached 85%, but what is the reason for the remaining 15% not being complete?  Please describe this in your consideration to the extent possible | Thank you for this remark. 15% incompleteness translates to n=3 missing/invalid data sets. A variety of reasons might be causative, for example: administration-related issues, user compliance, and data synchronization. In our discussion we elaborate on potential pitfalls when dealing with data completeness and the countermeasures we tried to apply during the conceptualization of this study and its methodology. Please see L. 461-467. |
| L247-248: You write 'No decline in data quality was observed', but how did you determine that there was no decline in quality? | We appreciate you pointing out this incoherence. This instructive criticism helped us refine and refocus our work. With regard to environmental monitoring, we decided to primarily focus on data completeness and removing outliers, if present, that the indoor hygrometer should not be able to measure. please see L. 267-269, 328-330. |
| L249-250: It says that the indoor WBGT was higher than the outdoor WBGT, but normally, the WBGT is thought to be higher outdoors, where the black globe temperature is higher. Please describe in the discussion section any possible reasons why the indoor WBGT was higher this time. | Thank you, your perceptive remarks have been integral to refining our work. We considered this point and clarified it in our updated discussion section, please see L. 539-546. |
| In addition, different methods are used to measure indoor and outdoor WBGT. These differ from the methods specified in ISO 7243, so is it possible to compare indoor and outdoor WBGT in this situation? Please consider this point as well | Thank you for bringing this to our attention. We acknowledge this issue and after careful consideration we decided to not compare outdoor and indoor WBGT directly, as it also does not really fall within the original scope of this work. Additionally, we updated our discussion on the issue of WBGT estimation methodology. Please see L. 537-535. |
| L314-318: Did the tendency to make negative statements about receptivity differ depending on the type of wearable device? Please describe this to the extent possible, as there is a possibility that receptivity will differ depending on the type of device. If you did not obtain any data, please state this in the discussion. | Thank you very much for the suggestion. Indeed, we did collect data reflecting the differences in receptivity towards different devices. We now included a summary of the data in our results section, please see L. 400-411. |
| L326-327: The same applies here as above. Were there any differences between the types of device? | Your valuable input is much appreciated. We did update the relevant section of the manuscript, as mentioned in the response above. Please refer to L. 400-411. |
